# Urinary microRNAs for the non-invasive diagnosis of endometriosis identified by next-generation sequencing and machine learning

**DOI:** 10.1186/s12958-025-01517-6

**Published:** 2026-01-07

**Authors:** Tomas Kupec, Julia Wittenborn, Chao-Chung Kuo, Laila Najjari, Rebecca Senger, Philipp Meyer-Wilmes, Elmar Stickeler, Jochen Maurer

**Affiliations:** 1 Department of Gynecology and Obstetrics, University Hospital RWTH Aachen, Aachen, Germany; 2 Genomics Facility, Interdisciplinary Center for Clinical Research (IZKF), University Hospital RWTH Aachen, Aachen, Germany

**Keywords:** Endometriosis, Chronic pelvic pain, Urine, miRNA, Liquid biopsy, Biomarker, RNA-based diagnostics

## Abstract

**Background:**

Endometriosis is a chronic gynecological disease associated with pain, infertility, and delayed diagnosis. Non-invasive biomarkers are urgently needed to facilitate earlier detection and reduce the reliance on diagnostic laparoscopy. MicroRNAs (miRNAs) are stable in body fluids and hold promise as diagnostic tools.

**Methods:**

In this pilot study, urine samples from 34 patients with histologically confirmed endometriosis and 18 control patients (laparoscopically confirmed absence of disease) were analyzed using next-generation miRNA sequencing. Differential expression analysis was performed with DESeq2. Feature selection applied variance filtering, univariate analysis, mutual information, and recursive feature elimination (RFE). The top 20 miRNAs were used to train four classification models: logistic regression, decision tree, random forest, and support vector machine (SVM). Model performance was evaluated by accuracy, precision, recall, F1-score, and area under the ROC curve (AUC).

**Results:**

Among all detected miRNAs, hsa-miR-10400-5p was significantly downregulated in endometriosis compared to controls (log₂ fold change − 2.70; adjusted *p* = 0.015). RFE identified 20 miRNAs, including hsa-mir-183, hsa-mir-500a, hsa-miR-3184-5p, hsa-miR-151b, and hsa-mir-196a-1, as the most informative for classification. The random forest model achieved the best performance (AUC = 0.91; accuracy and F1-score = 0.81), outperforming logistic regression (AUC = 0.83) and SVM (AUC = 0.81). Several identified miRNAs have been previously implicated in endometriosis pathogenesis, and we additionally identified hsa-miR-10400-5p as a significantly downregulated and previously unreported biomarker candidate, representing a novel finding with potential diagnostic relevance.

**Conclusions:**

Urinary miRNA profiling, combined with machine learning, shows promise as a completely non-invasive approach for the diagnosis of endometriosis. The identified miRNA signature, particularly the novel hsa-miR-10400-5p, warrants validation in larger, independent cohorts to confirm its clinical utility and potential to reduce diagnostic delays.

**Supplementary Information:**

The online version contains supplementary material available at 10.1186/s12958-025-01517-6.

## Introduction

Endometriosis is a chronic disease primarily affecting women of reproductive age, characterized by the presence of endometrial-like tissue outside the uterine cavity. The most frequent location is the peritoneum, but lesions may also occur on the ovaries, intestines, or uterine ligaments. The disease often results in chronic pelvic pain and infertility. Endometriosis affects approximately 10% of women of reproductive age [1]. However, many cases remain undiagnosed or unreported, leading to an underestimation of its true prevalence. Although awareness has increased in recent years, the time between symptom onset and definitive diagnosis can still span up to 10 years, with diagnosis often occurring only during infertility evaluations [2, 3].

Until a few years ago, diagnostic laparoscopy was considered the gold standard for confirming endometriosis. This led to many unnecessary surgical interventions in young women. Recently, this paradigm has been challenged: with a thorough clinical history, gynecological examination, and transvaginal ultrasound, a working diagnosis can now often be made and appropriate treatment initiated without the need for surgery [4, 5].

Endometriosis has a high prevalence and a substantial negative impact on quality of life [6, 7]. It also represents a major economic burden due to delayed diagnosis and inadequate treatment, resulting in repeated surgeries, hospitalizations, unsuccessful fertility treatments, work absenteeism, and disruptions to education and training [8]. There is a strong need for early, non-invasive diagnostic tools - yet such screening methods are currently lacking.

In 2022, Bendifallah et al. [9] developed a saliva-based miRNA panel that demonstrated promising diagnostic performance in detecting endometriosis non-invasively. However, external validation studies are still needed to confirm its clinical utility.

MicroRNAs (miRNAs) are small non-coding RNAs involved in post-transcriptional regulation of gene expression. They have already shown diagnostic and prognostic relevance in various cancers and chronic non-malignant conditions [10, 11]. Importantly, their remarkable stability in body fluids, even under conditions that typically degrade RNA, further supports their potential as robust non-invasive biomarkers [12].

The aim of our study is to investigate the potential of urine, an easily accessible and completely non-invasive body fluid, as a source for miRNA-based biomarkers in endometriosis. Beyond its accessibility, urine offers several analytical advantages over other non-invasive biofluids. Urinary miRNAs display remarkable stability under varying storage conditions [13], and urine generally contains fewer confounding cellular or protein contaminants, reducing the risk of pre-analytical artefacts commonly observed in serum due to haemolysis or platelet activation [14, 15]. In comparative studies, urine-derived miRNAs have demonstrated larger fold-changes and higher diagnostic accuracy than serum miRNAs, supporting their suitability as a biomarker matrix [16]. Urine samples from patients with histologically confirmed endometriosis were analyzed using next-generation miRNA sequencing. Our objective was to identify distinct urinary miRNA signatures that may be harnessed for diagnostic purposes. Various machine learning techniques were applied to assess the discriminatory power of these miRNAs and to support the development of a reliable, non-invasive diagnostic approach.

## Materials and methods

### Patient selection

This study analyzed data from 52 patients who presented with lower abdominal pain or clinical suspicion of endometriosis at the Endometriosis Centre of RWTH Aachen University Hospital between 12/2021 and 08/2023. Of these, 34 patients were diagnosed with histologically confirmed endometriosis, while 18 patients in whom endometriosis was ruled out served as the negative control group.

Before the clinical examination, all patients completed a standardized medical history questionnaire in the waiting area to document demographic characteristics and self-reported symptoms. The diagnosis of endometriosis was established in accordance with the current ESHRE guidelines [5], based on a structured gynaecological examination and transvaginal ultrasound, both conducted by a senior consultant specialized in endometriosis care. Each consultation also included individualized treatment planning, discussing therapeutic options such as hormonal therapy, surgical intervention, analgesic management, or reproductive assistance, as well as comprehensive counselling and patient support services.

Patients with adenomyosis uteri, uterine fibroids, inflammatory bowel disease, or other chronic inflammatory or gynaecological conditions were excluded based on a comprehensive clinical evaluation and detailed medical history. Eligibility criteria comprised patients scheduled for laparoscopic surgery during the study period due to a clinical suspicion of endometriosis, primarily based on symptoms of lower abdominal pain. All procedures were performed by one of three senior surgeons with extensive expertise in endometriosis care at the specialized Endometriosis Centre. During laparoscopy, a systematic inspection of the entire abdominal cavity was conducted, and any suspicious lesions were excised and submitted for histopathological evaluation. The diagnosis of adenomyosis uteri was assessed intraoperatively and supplemented by transvaginal ultrasound (TVUS), applying the criteria described by Harmsen et al. [17] patients in the endometriosis-positive group had histologically confirmed disease. The control group comprised patients who underwent surgery for suspected endometriosis but in whom both endometriosis and adenomyosis uteri were excluded based on intraoperative findings and histology.

The severity and localization of endometriotic lesions were classified intraoperatively using the #ENZIAN classification system, which provides a standardized framework for describing the extent and distribution of endometriosis [18].

All participants provided written informed consent prior to inclusion in the study. The study was conducted in accordance with the ethical principles outlined in the Declaration of Helsinki. Ethical approval was obtained from the Independent Ethics Committee of the Faculty of Medicine at RWTH Aachen University (approval number 206/09).

### Urine sample preparation

Before the surgery, a total of 52 urine samples were collected for analysis. Samples from all patients were centrifuged at 944 × g for 10 min at room temperature to separate the cellular pellet from the cell-free supernatant. Both fractions were stored separately at − 80 °C on the same day after registration of the data in the biobank of RWTH University hospital Aachen and labelling of the samples with a pseudonym until further processing.

For miRNA extraction, a minimum of 7 ml of cell-free supernatant was required. From each sample, 4 ml of the supernatant was used for RNA isolation, while the remaining volume was retained as backup. The miRNA was extracted from each urine sample using the miRNeasy Urine Kit (Qiagen, Hilden, Germany) following the manufacturer’s protocol.

### MiRNA sequencing and statistical analysis

Sequencing libraries were prepared using the QIAseq miRNA UDI Library Kit (Qiagen, Hilden, Germany) in accordance with the manufacturer’s protocol. For each sample, 4 µl of RNA input derived from biofluids was used, supplemented with 1 µl of synthetic spike-in miRNAs from the QIAseq miRNA Library QC Kit, serving as an internal quality control. Library quality was assessed using either a Bioanalyzer or TapeStation system (both Agilent, Waldbronn, Germany), and library concentration was quantified with a Quantus fluorometer (Promega, Madison, WI, USA). Sequencing was performed on an Illumina NextSeq 500 platform (Illumina, San Diego, CA, USA) using 72 bp single-end reads. The average sequencing depth was approximately five million reads per sample.

FASTQ files were generated using *bcl2fastq* (Illumina). To ensure reproducibility and standardization, all samples were processed using the publicly available nf-core/smRNAseq pipeline (version 2.3.0) [19], implemented in Nextflow version 23.10.1 [20] and executed via Docker version 24.0.2 with minimal configuration. Downstream analyses were conducted using custom scripts in R version 4.3.3, employing the DESeq2 framework (version 1.38.3) for differential expression analysis [21].

All samples from 34 patients with histologically confirmed endometriosis and 18 control patients were included in the downstream analyses. After normalization of read counts using the DESeq2 framework, five-fold cross-validation was applied to ensure robust performance evaluation. Our analytical strategy followed a two-step approach: first, machine learning models, including logistic regression, decision trees, random forest, and support vector machines (SVM), were trained on the full set of over 4,000 detected miRNAs to capture broad expression patterns and interactions. Second, model-based feature selection methods were applied to identify the most informative miRNAs contributing to classification accuracy. Data analysis and visualization were conducted using Python packages including *scikit-learn*, *numpy*, *pandas*, *matplotlib*, and *seaborn*, enabling a comprehensive exploration of the diagnostic potential of urinary miRNA profiles.

### Preprocessing

The raw dataset, comprising 4,285 microRNA (miRNA) features across 52 urine samples (34 endometriosis-positive and 18 endometriosis-negative), was preprocessed prior to statistical and machine learning analyses. Data were organized with features as rows and samples as columns, ensuring unique identifiers for both. Only numerical expression values were retained. Features with zero counts across all samples (*n* = 206) were excluded. Outlier detection using z-scores identified multiple samples with extreme expression values; these were retained for downstream analyses to preserve dataset integrity. The final dataset contained 4,079 miRNAs, which were subsequently standardized for dimensionality reduction and classification tasks.

### Feature selection

Feature selection was performed in multiple steps to reduce dimensionality and enhance model performance. Initially, features with zero variance were removed using the VarianceThreshold method, eliminating 427 features from the dataset. Univariate feature selection was then applied to evaluate each feature’s individual relationship with the target variable, retaining the top-ranked features. Subsequently, mutual information–based selection was employed to capture both linear and non-linear dependencies between features and the outcome, further reducing the feature set. Finally, recursive feature elimination (RFE) with the chosen estimator was used to iteratively remove the least relevant features, resulting in a final subset of 20 features for model development.

### Model selection

For model selection, the dataset containing the 20 selected features was randomly divided into a training set (*n* = 31 /*n* = 21 positive, *n* = 10 negative) and a test set (*n* = 21/*n* = 14 positive, *n* = 7 negative). The training set was used to develop and tune the classification models, while the independent test set was reserved for final performance evaluation. To ensure robust estimation of model performance and reduce overfitting risk, k-fold cross-validation was applied to the training set, with the same fold partitions used consistently across all candidate models. Separate scripts were used to train each model type, allowing for reproducibility and independent optimization. The final model was selected based on cross-validated performance metrics, and its generalization capability was subsequently assessed on the held-out test set.

### Model evaluation

Model evaluation was performed using the independent test set to assess the generalization performance of the final selected classifier. Performance metrics included accuracy, precision, recall, F1-score, and the area under the receiver operating characteristic curve (AUC-ROC), providing a comprehensive assessment of both overall and class-specific predictive capability. In addition, confusion matrices were generated to visualize classification outcomes and identify potential misclassification patterns. All metrics were computed using standardized functions from the scikit-learn library to ensure reproducibility.

Statistical analyses were performed using GraphPad Prism version 10 (GraphPad Software, San Diego, CA, USA). Student’s *t*-test was applied to assess statistically significant differences between groups.

No generative AI or AI-assisted technologies were used in the writing or preparation of this manuscript. All content was generated exclusively by the authors, who assume full responsibility for its accuracy and integrity.

## Results

The mean age of patients with endometriosis was 28.1 years (SD = 7.5; 95% CI: 25.5–30.7), compared to 26.6 years (SD = 6.1; 95% CI: 23.5–29.6) in the control group (*p* = 0.430). Based on intraoperative assessment and classification according to the #ENZIAN system, most lesions were located on the peritoneum (*#ENZIAN P*, *n* = 29), followed by the ovaries (*#ENZIAN O*, *n* = 6) and fallopian tubes (*#ENZIAN T*, *n* = 2). Deep infiltrating endometriosis (DIE) was diagnosed in 17 patients. Among these, the most frequently affected compartments were the uterosacral ligaments or parametrium (*#ENZIAN B*, *n* = 12) and the rectovaginal septum or vagina (*#ENZIAN A*, *n* = 5). Less commonly involved sites included the bladder (*#ENZIAN FB*, *n* = 2) and the intestine (*#ENZIAN FI*, *n* = 1). No cases of DIE involving the rectum or sigmoid colon (*#ENZIAN C*) were identified in this cohort. The characteristics of the patients included in the study are presented in Table 1.

**Table 1:** Baseline demographic and clinical characteristics of patients with endometriosis and the control group, including age, BMI, infertility history, pelvic pain symptoms, medical treatments, and #ENZIAN classification. Data are presented as mean (SD) or number (%)

Location of endometriosis diagnosis (#ENZIAN)	Number of Patients *n*	
#ENZIAN P (Peritoneal)	29	
#ENZIAN O (Ovary)	6	
#ENZIAN T (Tube)	2	
Deep infiltrating endometriosis	17	
#ENZIAN A	5	
#ENZIAN B	12	
#ENZIAN C	0	
#ENZIAN FB	2	
#ENZIAN FI	1	
	Patients with endometriosis	Control group
Age/years (Mean; SD)	28.10 (7.46)	26.6 (6.18)
BMI (Mean; SD)	23.87 (9.38)	22.64 (8.32)
Infertility (*n*)		
Yes	8 (24%)	7 (39%)
No	26 (76%)	11 (61%)
Dysmenorrhoe (*n*)	34 (100%)	18 (100%)
Dyspareunia (*n*)	12 (35%)	6 (33%)
Dyschesia (*n*)	8 (24%)	4 (22%)
Dysuria (*n*)	7 (21%)	5 (28%)
Medical treatments (*n*)		
Hormonal therapy	21 (62%)	11 (61%)
Analgesia	7 (21%)	5 (28%)

### MiRNA differential expression

Among all detected miRNAs, hsa-miR-10400-5p was significantly downregulated in the endometriosis group compared to controls (Table 2), with a log₂ fold change of − 2.70, a base mean expression of 37.42, and a Wald test statistic of − 4.63 (adjusted *p* = 0.015).

**Table 2:** Differential expression of hsa-miR-10400-5p

Gene Name	Base Mean	Log₂ Fold Change	lfcSE	Statistic	*p*-value	Adjusted *p*-value	Significance
hsa-miR-10400-5p	37.42	-2.70	0.58	-4.63	3.71 × 10⁻⁶	0.015	Significant

### Top 20 MiRNAs

**Fig. 1:**
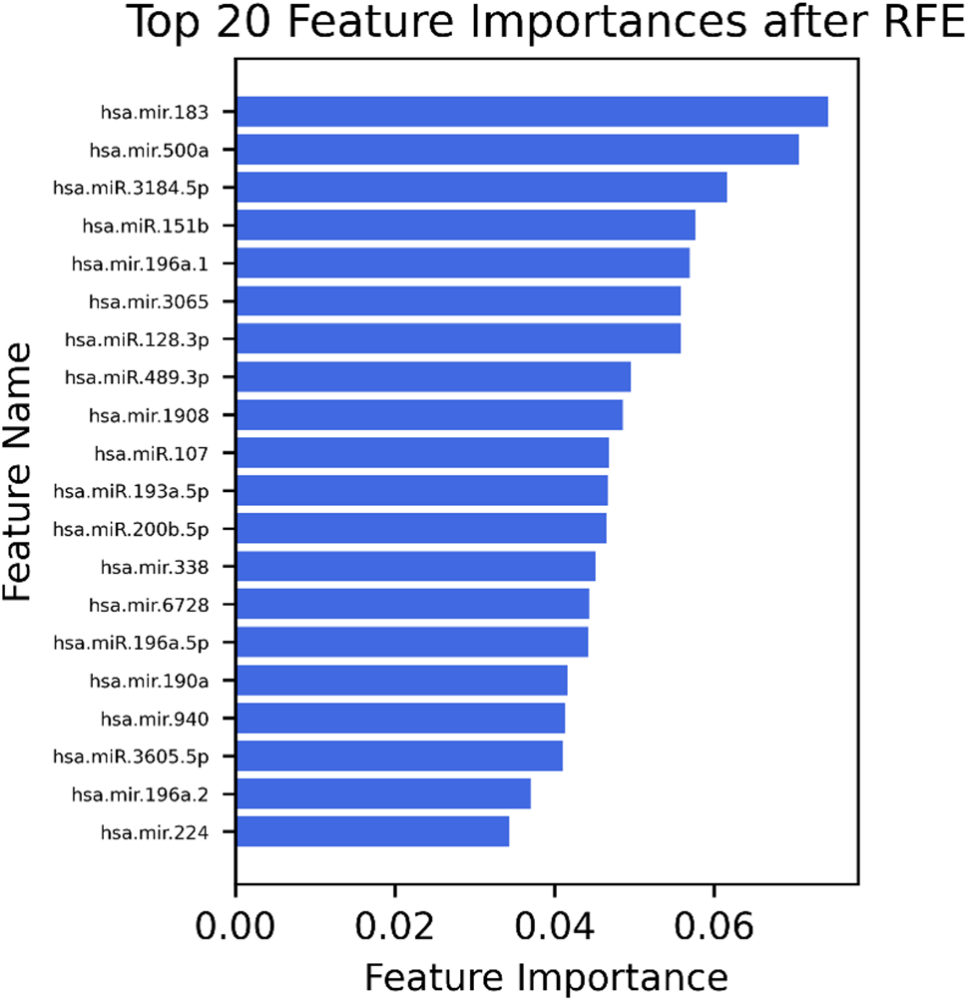
Top 20 urinary microRNAs selected by recursive feature elimination (RFE). Bar plot showing the 20 most informative miRNAs for distinguishing patients with histologically confirmed endometriosis from those without disease. The x-axis indicates the relative feature importance score, while the y-axis lists the corresponding miRNA identifiers. Higher values reflect a greater contribution of each miRNA to the classification model

Following the final step of (RFE), the top 20 (Fig. 1) most relevant miRNAs were identified: hsa-mir-183, hsa-mir-500a, hsa-miR-3184-5p, hsa-miR-151b, hsa-mir-196a-1, hsa-mir-3065, hsa-miR-128-3p, hsa-miR-489-3p, hsa-mir-1908, hsa-miR-107, hsa-miR-193a-5p, hsa-miR-200b-5p, hsa-mir-338, hsa-mir-6728, hsa-miR-196a-5p, hsa-mir-190a, hsa-mir-940, hsa-miR-3605-5p, hsa-mir-196a-2, and hsa-mir-224. These features represent the most informative biomarkers for distinguishing between endometriosis and control groups in urine samples. Detailed differential expression statistics for these top 20 candidate miRNAs, including fold changes and adjusted p-values, are provided in Supplementary Table S1.

### Conclusion of methodological evaluation

**Fig. 2:**
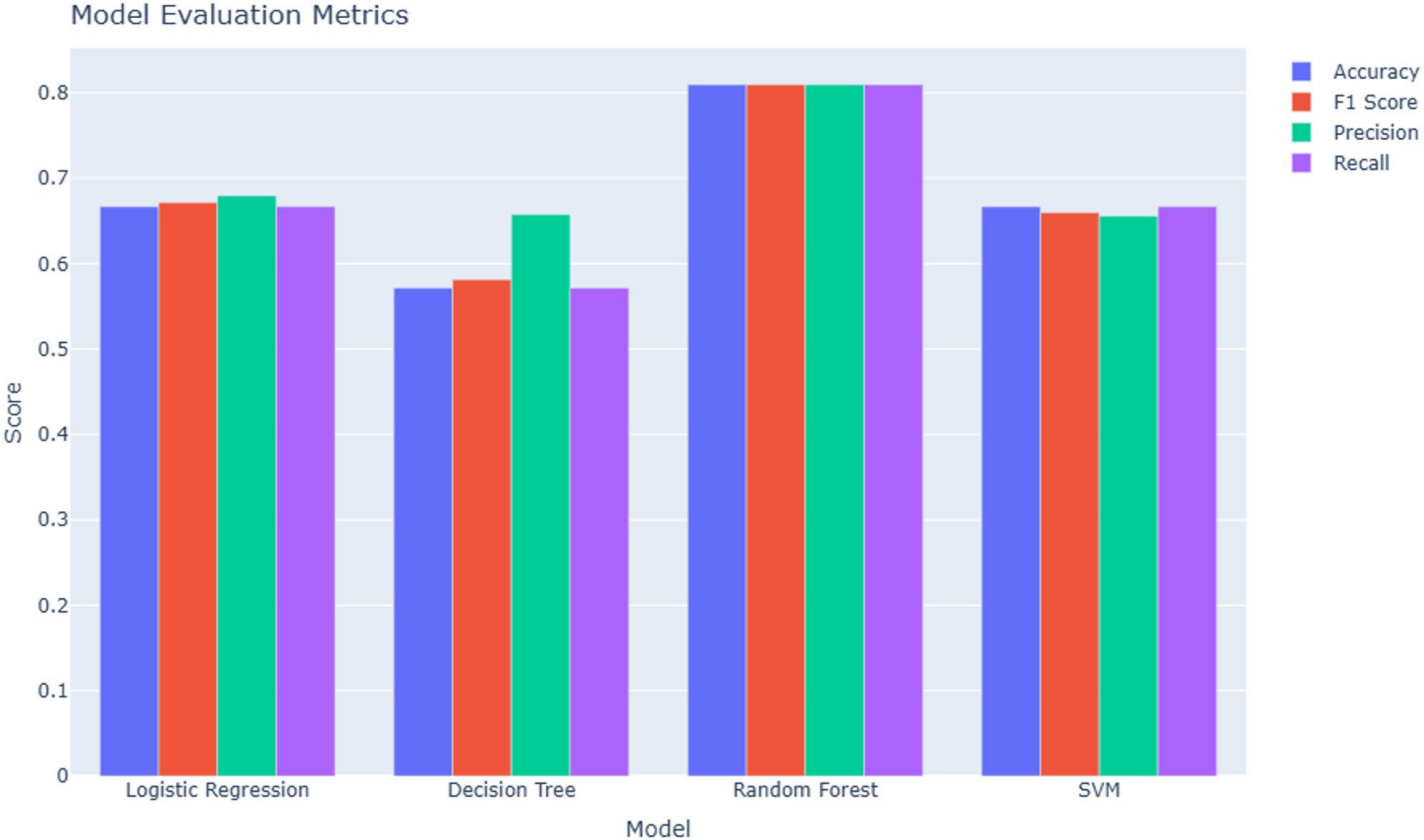
Performance comparison of machine learning models for urinary miRNA-based classification of endometriosis. Bar plot illustrating the evaluation metrics of four supervised learning algorithms (Logistic Regression, Decision Tree, Random Forest, and Support Vector Machine). Model performance was assessed by accuracy, precision, recall, and F1 score. The Random Forest model outperformed the other classifiers across all metrics, demonstrating the strongest ability to discriminate between endometriosis and control samples

The RFE identified the top 20 most informative features for model development. These selected features were used to train four supervised classification algorithms: logistic regression, decision tree, random forest, and support vector machine (Fig. 2). Among these, the random forest model achieved the highest performance, with an accuracy, precision, recall, and F1-score of 0.81 each, outperforming logistic regression (accuracy 0.67, F1-score 0.67), SVM (accuracy 0.67, F1-score 0.66), and decision tree (accuracy 0.57, F1-score 0.58).

**Fig. 3:**
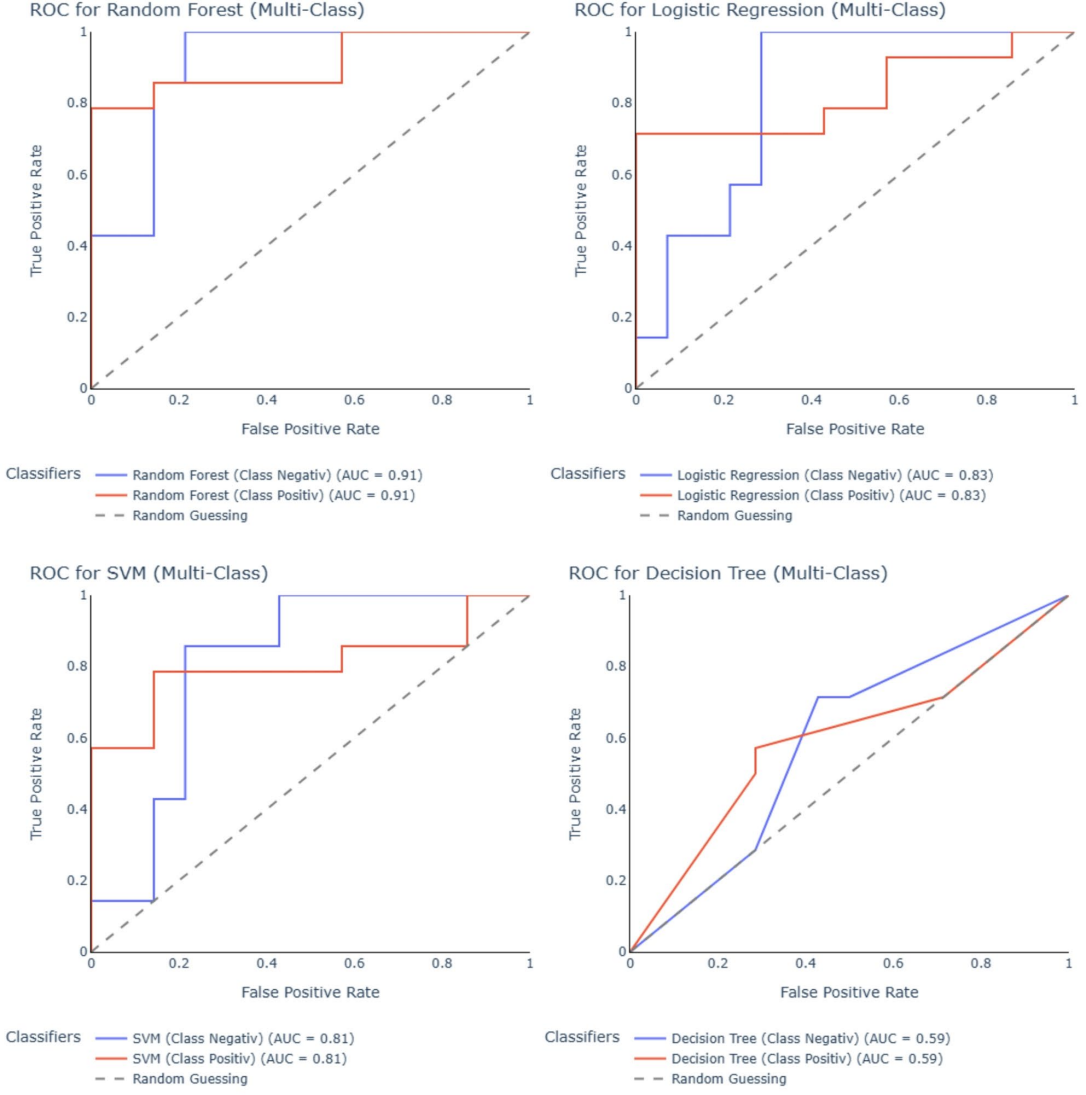
Receiver operating characteristic (ROC) curves of machine learning models for urinary miRNA-based classification of endometriosis. ROC curves display the discriminatory performance of four classifiers: Random Forest, Logistic Regression, Support Vector Machine (SVM), Decision Tree. Curves are shown for endometriosis-positive (red) and endometriosis-excluded (blue) classes, with the diagonal dashed line representing random guessing. Area under the curve (AUC) values are indicated for each class. The Random Forest model achieved the highest overall discrimination (AUC = 0.91), followed by Logistic Regression (AUC = 0.83), SVM (AUC = 0.81), and Decision Tree (AUC = 0.59)

ROC curve analysis further confirmed the superior discriminative performance of the random forest model (AUC = 0.91 for both classes), followed by logistic regression (AUC = 0.83), SVM (AUC = 0.81), and decision tree (AUC = 0.59) (Fig. 3).

## Discussion

In this pilot study, we investigated urinary miRNA profiles as a novel, completely non-invasive diagnostic approach for endometriosis. Our results demonstrate that specific urinary miRNA signatures can effectively distinguish patients with histologically confirmed endometriosis from controls, with the random forest model achieving the highest classification performance (AUC = 0.91; accuracy and F1-score = 0.81). Notably, pathway-driven feature selection through recursive feature elimination identified 20 miRNAs, including hsa-mir-183, hsa-mir-500a, hsa-miR-3184-5p, hsa-miR-151b, and hsa-mir-196a-1, as the most informative for classification.

Importantly, the identification of hsa-miR-10400-5p as significantly downregulated in the endometriosis group (log₂ fold change − 2.70; base mean 37.42; adjusted *p* = 0.015) supports the biological plausibility of urinary miRNAs as potential biomarkers, given the established role of miRNAs in regulating inflammatory and hormonal pathways involved in endometriosis pathophysiology. To our knowledge, this is the first report associating hsa-miR-10400-5p with endometriosis. Although its biological functions remain poorly described, preclinical studies suggest potential roles in intracellular trafficking and immune modulation through regulation of targets such as ARPC1A, RAB10, VPS35, and RhoA. A particularly relevant target is ARPC1A, a regulatory subunit of the actin-related protein 2/3 (ARP2/3) complex, which drives branched actin polymerization and regulates key processes such as cell migration, invasion, adhesion, and cytoskeletal remodelling. Dysregulation of ARP2/3-mediated actin dynamics has been associated with enhanced cellular motility and invasiveness mechanisms directly involved in the establishment and progression of ectopic endometrial lesions. Reduced expression of hsa-miR-10400-5p could therefore lead to increased ARPC1A activity, potentially promoting aberrant actin remodelling, altered behaviour of endometrial stromal cells, and enhanced tissue infiltration. This predicted interaction offers a biologically plausible mechanistic link between hsa-miR-10400-5p downregulation and pathways central to endometriosis pathophysiology, although functional validation remains necessary [22]. In human studies, it has been reported as downregulated in colorectal cancer cells following retinoid treatment and associated with enhanced apoptosis and reduced ER stress adaptation in osteosarcoma cells [23]. These observations suggest hsa-miR-10400-5p as a novel biomarker candidate warranting further investigation.

Several other miRNAs identified in our top 20 panel have been implicated in endometriosis pathogenesis. For example, hsa-mir-183 has been shown to be significantly downregulated in both ectopic and eutopic endometrial tissues compared to healthy controls; it promotes apoptosis and limits the invasiveness of endometrial stromal cells (ESCs), consistent with a tumor-suppressive role in endometriosis [24, 25]. Mechanistically, hsa-mir-183 suppresses cell migration and invasion by downregulating Ezrin and inhibiting Rho/ROCK signalling-pathways implicated in cytoskeletal remodelling and disease progression [26]. Similarly, hsa-miR-500a has been found over ten-fold upregulated in serum of patients with endometriosis in microarray analyses [27]. While hsa-miR-3184-5p has not yet been linked to endometriosis, it exerts tumor-suppressive effects in ovarian and breast cancers via modulation of XBP1/AKT/STAT3, FOXP4, and EMT-related signalling [28–30]. Hsa-miR-151b, though not previously associated with endometriosis, has been implicated in neurological processes and may interact with GPER1, a receptor relevant to neurobiological regulation [31, 32]. Finally, hsa-miR-196a is upregulated in eutopic endometrium of women with minimal or mild endometriosis, promoting MEK/ERK signalling, suppressing progesterone receptor expression, and impairing decidualization in endometrial stromal cells [33].

These findings expand upon earlier reports, including the saliva and serum-based miRNA panels of Bendifallah et al. [9] and Kupec et al. [34], and confirm that urine, an easily accessible, non-invasive biofluid, can harbor disease-specific miRNA signatures. While our cohort size was limited, the reproducibility of selected miRNAs across multiple machine learning algorithms underscores their potential for integration into clinical screening protocols pending validation in larger cohorts.

Several previous studies have investigated the role of circulating, endometrial, and lesion-specific miRNAs in the early diagnosis of endometriosis. Cho et al. reported differential expression of several circulating miRNAs in the serum of women with endometriosis, including let-7 family and hsa-miR-3613-5p [35]. Cosar et al. [27] identified in serum hsa-miR-125b-5p as a particularly promising single marker, with diagnostic performance further improved when combined with hsa-miR-451a and hsa-miR-3613-5p. Moustafa et al. [36] confirmed in independent cohorts that serum miRNA signatures can achieve high diagnostic accuracy (AUC > 0.9). In addition, Saare et al. identified lesion-specific miRNAs through high-throughput sequencing that could distinguish endometriotic lesions from surrounding tissue for the first time [37].

A major strength of this study is the rigorous selection of a well-defined cohort of patients with chronic pelvic pain and suspected endometriosis, all of whom underwent laparoscopic evaluation. This ensured histological confirmation in all cases of endometriosis and surgical exclusion in all controls, enabling accurate case–control classification. The use of next-generation sequencing combined with advanced feature selection and machine learning provided a robust, unbiased approach to biomarker discovery.

This pilot study has several limitations. The cohort size was relatively small (*n* = 52), which increases the risk of overfitting and may inflate the diagnostic performance of the machine learning models. The absence of an independent external validation cohort further limits generalizability and underscores the need for larger, multicentre studies. In addition, clinical variables, such as menstrual cycle phase, ongoing hormonal or other medical treatments, and patient ancestry, were not systematically recorded, although these factors may influence urinary miRNA expression through hormonal, metabolic, immune, or genetic mechanisms. Their absence restricts the interpretation of potential confounders. Moreover, the biological functions of several identified miRNAs, particularly hsa-miR-10400-5p, remain insufficiently characterized and require functional validation. While some of the miRNAs identified in our panel (e.g., hsa-miR-183, hsa-miR-500a) overlap with markers described in serum- or tissue-based studies, the identification of hsa-miR-10400-5p as a significantly downregulated and previously unreported miRNA in endometriosis represents a novel biomarker candidate that warrants further investigation. Despite these limitations, our findings support the feasibility of urinary miRNAs as completely non-invasive biomarkers for endometriosis and provide a strong rationale for validation in larger, well-phenotyped cohorts.

From a clinical perspective, urinary miRNA testing could be integrated into several points of the diagnostic workflow for women with suspected endometriosis. First, as a completely non-invasive and easily repeatable assay, it could serve as an early screening tool for patients presenting with chronic pelvic pain, helping identify those who may benefit from expedited specialist evaluation. Second, urinary miRNA profiles may function as a triage tool before diagnostic laparoscopy, potentially reducing unnecessary surgical procedures by better stratifying patients according to their likelihood of having endometriosis. Third, urinary miRNA testing may complement imaging modalities such as transvaginal ultrasound, particularly in cases with inconclusive findings. In this context, a validated urinary miRNA signature could support a more accurate, multimodal diagnostic approach and contribute to a more robust diagnostic process.

## Conclusion

This pilot study demonstrates the potential of urinary miRNA profiling as a completely non-invasive diagnostic approach for endometriosis. We identified hsa-miR-10400-5p as a significantly downregulated biomarker in patients with histologically confirmed disease, along with a panel of 20 highly informative miRNAs identified through machine learning. This panel included both previously reported miRNAs, such as hsa-mir-183, and novel candidates, such as hsa-miR-151b, not yet described in this context. Given the relatively small cohort size and the lack of an independent external validation dataset, the diagnostic performance observed in this study—particularly the high AUC values—should be interpreted with caution, as such results may be overestimated in small pilot cohorts. While further validation is required, these results provide a strong rationale for incorporating urinary miRNA analysis into non-invasive diagnostic strategies aimed at reducing diagnostic delays and unnecessary surgeries in endometriosis care.

## Supplementary Information

Supplementary Material 1.

## Data Availability

The datasets generated during the current study are available from the corresponding author on reasonable request.
