# Supplementary material for: Urinary microRNAs for the non-invasive diagnosis of endometriosis identified by next-generation sequencing and machine learning
Source: Reprod Biol Endocrinol. 2026 Jan 7;24:4. doi: 10.1186/s12958-025-01517-6 (PMC12781734; doi:10.1186/s12958-025-01517-6)
Supplement: Supplementary file 1 — Supplementary Material 1. [file 12958_2025_1517_MOESM1_ESM.docx]

| **Table S1. Differential expression statistics for the top 20 miRNAs identified in the study cohort.** | | | | | | | |
| --- | --- | --- | --- | --- | --- | --- | --- |
| Gene Name | Base Mean | Log₂ Fold Change | lfcSE | Statistic | p-value | Adjusted p-value | Significance |
| hsa-mir-183 | 7.40 | 0.83 | 0.28 | 2.99 | 0.003 | 0.607 | Non-sig. |
| hsa-mir-500a | 5.41 | -0.68 | 0.27 | -2.56 | 0.010 | 0.999 | Non-sig. |
| hsa-miR-3184-5p | 1.10 | -0.95 | 0.44 | -2.19 | 0.029 | 0.999 | Non-sig. |
| hsa-miR-151b | 2.93 | -0.68 | 0.37 | -1.86 | 0.063 | 0.999 | Non-sig. |
| hsa-mir-196a-1 | 45,74 | -0.24 | 0.20 | -1.21 | 0.226 | 0.999 | Non-sig. |
| hsa-mir-3065 | 3.63 | 0.92 | 0.34 | 2.67 | 0.008 | 0.999 | Non-sig. |
| hsa-miR-128-3p | 9.02 | -0.55 | 0.25 | -2.23 | 0.027 | 0.999 | Non-sig. |
| hsa-miR-489-3p | 22.89 | -0.20 | 0.31 | -0.65 | 0.513 | 0.999 | Non-sig. |
| hsa-mir-1908 | 12.93 | -1.67 | 0.61 | -2.72 | 0.007 | 0.971 | Non-sig. |
| hsa-miR-107 | 100.34 | -0.53 | 0.24 | -2.22 | 0.026 | 0.999 | Non-sig. |
| hsa-miR-193a-5p | 56.20 | -0.13 | 0.21 | -0.62 | 0.534 | 0.999 | Non-sig. |
| hsa-miR-200b-5p | 17.23 | 0.25 | 0.20 | 1.24 | 0.213 | 0.999 | Non-sig. |
| hsa-mir-338 | 3.01 | 0.86 | 0.35 | 2.44 | 0.015 | 0.999 | Non-sig. |
| hsa-mir-6728 | 0.38 | -1.53 | 1.04 | -1.47 | 0.142 | 0.999 | Non-sig. |
| hsa-miR-196a-5p | 305.93 | -0.35 | 0.20 | -1.77 | 0.076 | 0.999 | Non-sig. |
| hsa-mir-190a | 2.83 | 1.34 | 0.52 | 2.57 | 0.010 | 0.999 | Non-sig. |
| hsa-mir-940 | 3.86 | 0.37 | 0.49 | 0.74 | 0.456 | 0.999 | Non-sig. |
| hsa-miR-3605-5p | 0.53 | -0.99 | 0.61 | -1.62 | 0.104 | 0.999 | Non-sig. |
| hsa-mir-196a-2 | 203.27 | -0.27 | 0.21 | -1.26 | 0.206 | 0.999 | Non-sig. |
| hsa-mir-224 | 5.18 | 0.95 | 0.40 | 2.38 | 0.017 | 0.999 | Non-sig. |
